# Differential Immunoreactivity to Bovine Convalescent Serum Between *Mycoplasma bovis* Biofilms and Planktonic Cells Revealed by Comparative Immunoproteomic Analysis

**DOI:** 10.3389/fmicb.2018.00379

**Published:** 2018-03-05

**Authors:** Shengli Chen, Huafang Hao, Ping Zhao, Wenheng Ji, Mingxia Li, Yongsheng Liu, Yuefeng Chu

**Affiliations:** State Key Laboratory of Veterinary Etiological Biology, Lanzhou Veterinary Research Institute, Chinese Academy of Agricultural Sciences, Lanzhou, China

**Keywords:** biofilms, *Mycoplasma bovis*, immunoprotein, immunoproteomics, planktonic cells

## Abstract

*Mycoplasma bovis* is a major bovine pathogen that causes considerable economic losses in the cattle industry worldwide. Moreover, *M. bovis* biofilm can persist in the environment and its host. To date, *M. bovis* biofilm antigens recognized by bovine convalescent sera and their comparison with planktonic cells have not yet been explored. This study utilized an immunoproteomic approach using two-dimensional electrophoresis, immunoblotting using convalescent bovine serum, and subsequent matrix-assisted laser desorption/ionization time-of-flight tandem mass spectrometry (MALDI-TOF/TOF MS) to identify the immunoreactive proteins expressed in biofilm- and planktonic-grown *M. bovis* strain 08M. Results showed that *M. bovis* biofilms and planktonic cells demonstrate differential immunoreactivity to bovine convalescent serum for the first time. A total of 10 and 8 immunoreactive proteins were identified for biofilms and planktonic cells, respectively. To our knowledge, a total of 12 out of 15 had not been reported as immunoreactive proteins in *M. bovis*, and six were specific to *M. bovis* biofilms. Three proteins, namely, endoglucanase, thiol peroxidase, and one putative membrane protein, that is, mycoplasma immunogenic lipase A, were identified in planktonic cells and biofilms. Most of the identified proteins were cytoplasmic proteins that were mainly involved in transport and metabolism. Moreover, ATP binding, oxidoreductase activity, and GTP binding were their most representative molecular functions. DnaK and Tuf appeared to be the most interactive immunoreactive agent among the identified proteins. Furthermore, six proteins had potential as serodiagnostic antigens. These data will be helpful to improve our current understanding on the host response to *M. bovis* biofilms and planktonic cells, which may facilitate the development of novel molecular candidates of improved diagnostics and vaccines to prevent *M. bovis* infections.

## Introduction

*Mycoplasma bovis* is an important pathogen that can cause pneumonia, mastitis, arthritis, otitis, meningitis, and keratoconjunctivitis in cattle and result in considerable economic losses worldwide (Rosengarten and Citti, 1999; Nicholas et al., 2000; Nicholas and Ayling, 2003; Xin et al., 2008). This livestock pathogen is a member of the Mollicutes class. Many *M. bovis* strains can form biofilms, and the ability for biofilm formation varies among different strains (Mcauliffe et al., 2006). The biofilm growth provides *M. bovis* several advantages over its planktonic counterparts; these advantages include remarkable resistance to antimicrobials and persistence in the environment and host, which may result in the chronicity of a disease (Mcauliffe et al., 2006) and considerable difficulty of treatment. Biofilms are important in the pathogenesis of several chronic bacterial infections, and lead to persistent infections (Parsek and Singh, 2003). The growth of bacterium under biofilm conditions with increased drug resistance and virulence may be due to the altered metabolism and different protein expression profiles compared with those of planktonic cells (Sauer, 2003; Seneviratne et al., 2012).

Immunoproteins play a key role in host–bacteria interaction by eliciting host immunization and bacterial pathogenesis. Immunoproteomics has been used extensively to identify immunoproteins and virulence factors in several important livestock pathogenic mycoplasma species, such as *Mycoplasma mycoides* subsp. *mycoides* small-colony type (MmmSC) (Jores et al., 2009), *M. capricolum* subsp. *capripneumoniae* (Mccp) (Zhao et al., 2012), and *Mycoplasma mycoides* subsp. *capri* (Mmc) (Corona et al., 2013). Numerous studies investigated immunoproteins in different components of *M. bovis* in planktonic growth using immunoproteomic assays, in which many related proteins were identified. Variable surface lipoproteins, which vary in phase and size, are *M. bovis* immunogenic proteins that play a key role in evading the immune system of the host (Sachse et al., 1996, 2000). The conserved P26 (Sachse et al., 1993) and P48 lipoproteins (Robino et al., 2005), mycoplasma immunogenic lipase A (MilA) (Wawegama et al., 2014), and pMB67 (Behrens et al., 1996) are all surface proteins and are immunogenic. Several cytoplasmic proteins, including heat-shock protein 60 and glyceraldehyde-3-phosphate dehydrogenase, are *M. bovis*-immunogenic proteins. Sun et al. (2014) identified 19 immunoproteins from *M. bovis* strain PD via immunoproteomics with four naturally infected positive sera of *M. bovis*. Khan et al. (2016) recently identified 39 immunogens from *M. bovis* strain HB0801 by using whole-cell protein and membrane fraction analysis; among these identified proteins, 32 have not been reported. To date, *M. bovis* biofilm antigens that are recognized by bovine convalescent sera have not been explored and compared with planktonic cells.

The present study utilized an immunoproteomics approach using 2-DE, immunoblotting, and matrix-assisted laser desorption/ionization time-of-flight tandem mass spectrometry (MALDI-TOF/TOF MS) to identify the antigen profiles of *M. bovis* biofilms and planktonic cells that react with bovine convalescent sera. Bioinformatic analysis and immunogenicity confirming analysis were further performed. These data are expected to improve our current understanding on the host immune response to *M. bovis* biofilms and develop novel serological diagnostic markers and vaccine candidates for *M. bovis* infection.

## Materials and Methods

### Ethics Statement

All animals were handled in strict accordance with the requirements of the Animal Ethics Procedures and Guidelines of the People’s Republic of China. All efforts were exerted to minimize the suffering of animals. The animal experimental protocol was reviewed and approved by the Animal Ethics Committee of Lanzhou Veterinary Research Institute, Chinese Academy of Agricultural Sciences (Permit No. LVRIAEC2010-007, LVRIAEC2015-009).

### Mycoplasma Isolate and Growth Conditions

*Mycoplasma bovis* strain 08M, which can form biofilms, was isolated from the pneumonia-infected lung of a calf in Ningxia province, China in 2008 (Chen et al., 2017). Biofilms were grown in 150 mm plates that contained 30 mL of PPLO broth (21 g/L PPLO broth, 1 g/L glucose, 2 g/L sodium pyruvate, 100 mL/L 25% yeast extract, 200 mL/L horse serum, pH 7.4–7.6), inoculated with a 1:10 dilution of a 20 h planktonic culture (about 7.40 × 10^8^ CFU/mL), and left at 37°C for 72 h without shaking. The supernatants were removed, and the plates were washed gently twice with PBS (0.01 M) at pH 7.2. Biofilms were detached by scraping, collected by centrifugation at 12,000 × *g* for 30 min, and resuspended in 1 mL of Tris-HCl (25 mM) at pH 7.2. Planktonic cells were grown in a 1 L bottle that contained 500 mL of PPLO broth at 37°C for 72 h without shaking. The cells were pelleted and washed, as described previously. The planktonic and biofilm cells examined in this study were in the stationary phase.

### Scanning Electron Microscopy Analysis

Scanning electron microscopy was conducted to observe the microstructure of the biofilms formed by *M. bovis* strain 08M on glass coverslips and *M. bovis* planktonic cells. Briefly, the biofilms on the glass coverslips were washed gently three times with PBS (0.01 M) at pH 7.2. Planktonic cells were collected by centrifugation at 12,000 × *g* for 30 min and washed three times with PBS (0.01 M) at pH 7.2. *M. bovis* biofilms and planktonic cells were fixed with 2.5% SEM-grade glutaraldehyde (Sigma-Aldrich, St. Louis, MO, United States), briefly rinsed with PBS twice for 5 min, washed with 4% sucrose, and dehydrated through a graded series of ethanol concentrations (30%, 50%, 70%, 80%, 90%, 95%, and 100%), with each series of ethanol concentrations lasting for 10 min. Finally, the fixed samples were dried by a Quorum K850 critical-point drying apparatus and sputter coated with gold palladium. Observations were performed at 20 kV with a FEI Inspect F SEM.

### Preparation of Bovine Antisera Against *M. bovis*

Convalescent sera against *M. bovis* 08M were prepared as followed: Three 6-month-old, clinically healthy calves free of *M. bovis* were infected with *M. bovis* 08M strain (1.0 × 10^9^ CCU/mL, 10 mL/calf, intratracheally). Bloods were collected from all calves on 0, 8, 17, 30, and 46 days after infection. Titers of the sera sample were evaluated by using a commercial *M. bovis* antibody ELISA Kit (Bio-X BIO K 302, Bio-X Diagnostics, Rochefort, Belgium). The pooled serum recovered from clinical illness with high titer (46 dpi, OD_450nm_ = 1.068) was used in the immunoblotting experiments. The pooled pre-infected serum (0 dpi, OD_450nm_ = 0.071) was used as a negative control.

Bovine-immune sera against *M. bovis* were prepared as described below: *M. bovis* 08M cells were grown in PPLO broth, collected, washed, and concentrated by PBS (0.15 M) at pH 7.2. The protein content was determined using the Bradford method to reach 0.46 and 1.48 mg/ml. Afterward, mycoplasma cells were inactivated with 4 mM binary ethyleneimine at 37°C for 24 h, following the addition of a final concentration of 4 mM Na_2_S_2_O_3_ at 37°C for 24 h, then emulsified with the same volumes of ISA 201 VG adjuvant (SEPPIC, France). Six 8-month-old, clinically healthy calves free of *M. bovis* were subcutaneously injected with 2 mL inactivated vaccine. A booster injection was administered 28 days after the initial immunization using the same method and doses. Sera samples were collected weekly from all calves from the time of initial immunization until day 28 after the booster injection. Titers of the sera sample were evaluated using a commercial *M. bovis* antibody ELISA Kit. Bovine antisera with over 2.0 ELISA titers were pooled and used for the Western blot analysis of the selected protein.

### Preparation of Whole Proteins From *M. bovis* Cells

Protein was extracted from three independent cultures of biofilms and planktonic cells as previously described (Carvalhais et al., 2015), with several modifications. Briefly, cell pellets of *M. bovis* biofilms and planktonic cultures were resuspended in SDT lysis buffer (4% SDS, 100 mM DTT, and 100 mM Tris-HCL). The cell suspension was vortexed, placed in a boiling water bath for 5 min, and mechanically broken using MP Biomedicals FastPrep-24^TM^ Instrument (6 m/s, 30 s per time, three times). Cell suspensions were sonicated on ice at 80 W for 10 cycles (10 s on, 15 s off), and placed in a boiling water bath for 5 min. Cell debris and unbroken cells were removed by centrifugation at 12,000 × *g* for 30 min at 25°C. The proteins in the supernatant were precipitated using 10 volumes of 10% chilled acetone at -20°C for 12 h. Precipitated protein was collected by centrifugation at 12,000 × *g* for 10 min at 4°C and washed two times with chilled acetone. The final pellet was air dried. The dried pellet was dissolved in 2D lysis buffer (8 M urea, 4% CHAPS, 40 mM Tris, 65 mM DTT, cocktail), incubated for 30 min at 25°C (vortexed every 10 min), and centrifuged at 12,000 × *g* for 20 min at 25°C. Prior to rehydration, the supernatant was treated with 2D Clean-up Kit (GE Healthcare) to remove contaminants that can interfere with electrophoresis. The protein content was determined by using the Bradford protein assay kit (Beijing Leagene Biotech, Beijing, China) according to the manufacturer’s instructions.

### 2-DE and Image Analysis

For the 2-DE, 200 μg of proteins were loaded onto analytical and preparative gels. Ettan IPGphor Isoelectric Focusing System (GE Amersham) and pH 3–10 IPG strips (13 cm, non-linear; GE Healthcare) were used for IEF. The IPG strips were rehydrated in 250 μL of rehydration buffer (8 M Urea, 2% CHAPS, 18 mM DTT, 0.5% IPG buffer, bromophenol blue trace), which contained the protein samples, for 30 min at room temperature. IEF was performed in the following steps: 30 V for 12 h, 500 V for 1 h, 1000 V for 1 h, 8000 V for 8 h, and 500 V for 4 h. After IEF, the IPG strips were equilibrated for 15 min in an equilibration buffer I (50 mM Tris-HCl pH 8.8, 6 M urea, 2% SDS, 30% glycerol, 1% DTT) and for another 15 min in an equilibration buffer II (50 mM Tris-HCl pH 8.8, 6 M urea, 2% SDS, 30% glycerol, 4% iodoacetamide). SDS-PAGE was conducted vertically in a Hofer SE 600 (Amersham Biosciences) using 12.5% polyacrylamide gels. The gels were then stained using modified silver staining methods that are compatible with subsequent mass spectrometric analysis (Yan et al., 2000). 2-DE was performed in triplicate for each growth condition.

The stained 2-DE gels were scanned with UMax Powerlook 2110XL (GE Amersham) and analyzed using ImageMaster^TM^ 2D Platinum 5.0 software (GE Healthcare, United States) according to the manufacturer’s instruction. The gels of *M. bovis* biofilms and planktonic cells were compared using ImageMaster^TM^ 2D Platinum 5.0 software (GE Healthcare, United States), as previously described (Wang et al., 2014). Briefly, the individual spots were quantified by calculating the relative spot volumes, which is the ratio of individual spot volume and total volume of all spots, and were expressed in volume percentage (vol.%). Changes in protein expression were calculated based on the overlapping measure ratios of corresponding spots by using ImageMaster^TM^ 2D Platinum 5.0 software according to the manufacturer’s instruction. Spots that showed volume alteration greater than 50% (fold change ≥ 1.5) at 95% confidence interval (Student’s *t*-test; *p* < 0.05) were considered to be statistically significant, and five selected differentially expressed protein spots were subjected to subsequent identification by mass spectrometry.

### Immunoblotting 2-DE Analysis

Immunoblotting analysis was conducted for each sample simultaneously using 2-DE analysis. The separated protein spots from 2-DE gels were transferred onto PVDF membrane (GE Healthcare, United States) using TE62 Tank Transfer Unit system (GE Healthcare, United States) at 100 V for 2 h. After blocking at room temperature for 2 h with 10% skimmed milk (Becton, Dickinson and Company, Franklin Lakes, NJ, United States) in TBST (0.5% v/v Tween) with gentle swinging, the membrane was incubated with the pooled convalescent sera against *M. bovis* at 4°C overnight. The membrane was washed three times with TBST buffer at 5 min each wash. Subsequently, the membrane was incubated with 1:8000 diluted HRP-conjugated goat anti-bovine IgG (Santa Cruz Biotech, United States) at room temperature for 1 h and washed five times with TBST buffer at 8 min each wash. Finally, the membranes were treated with Super Enhanced Chemiluminescent Substrate (ECL) Plus kit (Thermo Scientific, United States) to visualize the immunoreactive protein spots, according to the manufacturer’s instructions. Blots were scanned using Typhoon TMFLA 9500 (GE Amersham, United States). ImageMaster^TM^ 2D Platinum 5.0 software (GE Healthcare, United States) was used to match the spots on the membranes with their homologs in 2-DE gels stained with a modified silver staining method that was compatible with the subsequent mass spectrometric analyses. The pooled pre-infected serum of healthy calves served as negative controls in the 2-DE immunoblotting analysis. The process was performed in triplicate.

### Protein Identification by Mass Spectrometry

All of the immunoreactive proteins were selected and excised from 2D gels, which corresponded to the spots on the PVDF membranes and further in a gel digested with trypsin. MALDI-TOF-MS analysis was performed by using a MALDI-TOF/TOF instrument (5800 proteomics analyzer; Applied Biosystems) to identify proteins. Combined MS and MS/MS queries were conducted by using the Mascot search engine (Version 2.2; Matrix Science, Ltd.) on the database of UniProt *M. bovis* (downloaded on May 20, 2016; 1695 sequences) with the following parameter settings: Trypsin digestion, variable modification of oxidation (M), fixed modifications of carbamidomethyl (C), peptide mass tolerance for monoisotopic data of 100 ppm, one max missed cleavages, and MS/MS fragment tolerance of 0.4 Da. GPS Explorer protein with confidence index ≥ 95% (protein score C. I. %) was used for further manual validation.

### Bioinformatics Analysis

Gene Ontology^1^, Clusters of Orthologous Groups of proteins (COGs)^2^, and KEGG^3^ databases were used for functional annotation of the identified proteins. The online software PSORTb v.3.0^4^ was applied to predict the subcellular localization of identified proteins. Protein interaction analysis was conducted with STRING database^5^.

### SDS-PAGE and Western Blot Analysis of Recombinant Selected Proteins

Six identified proteins, namely, endoglucanase, GTPase Era (Era), hydrolases of the HAD superfamily protein, phosphotransacetylase (Pta_1), chaperone protein DnaK (DnaK), and energy-coupling factor transporter ATP-binding protein EcfA (EcfA), were selected to confirm their immunogenicity by using SDS-PAGE and subsequent Western blot analysis. The complete genes of these selected proteins with single nucleotide mutation in the UGA codon (UGA to UGG) were synthesized by Genecreate (Wuhan, China) and cloned into pET-30a(+) vectors (Novagen, Darmstadt, Germany). The constructs were confirmed by sequencing on ABI 3730XL. The recombinant proteins were expressed in *E. coli* BL21(DE3) cells (Novagen, Darmstadt, Germany), purified by NTA-NI (Genscript, Nanjing, China), and examined by SDS-PAGE. The immunogenicity of the proteins was confirmed by Western blot analysis. Briefly, purified proteins were transferred onto 0.45 μm PVDF membrane (Millipore, United States) using a fast semi-dry eBlot^TM^ protein transfer system (Genscript, Nanjing, China) for 7 min and blocked with 10% skimmed milk (Becton, Dickinson, and Company, Franklin Lakes, NJ, United States) in TBST (0.5% v/v Tween) with gentle swinging at 4°C overnight. After washing with TBST, the membrane was incubated with 1:100 dilution in the bovine-immune sera or convalescent sera against *M. bovis* for 1 h at room temperature. Afterward, the membrane was washed and incubated with 1:8000 diluted HRP-conjugated goat anti-bovine IgG (Santa Cruz Biotech, United States) for 1 h at room temperature. Finally, the membranes were washed and treated with Super ECL Plus kit (Thermo Scientific, United States) to visualize the immunoreaction according to the manufacturer’s instructions.

### Statistical Analysis

Statistical analysis was performed by using SPSS 15.0 (SPSS Inc., Chicago, IL, United States). Student’s *t*-test was used for statistical analysis. Mean and standard deviations (SD) were determined for the independent experiments and the results were expressed as mean ± SD. *p* < 0.05 was considered statistically significant.

## Results

### Scanning Electron Microscopy Analysis of Biofilm Formation of *M. bovis* 08M

**FIGURE 1:**
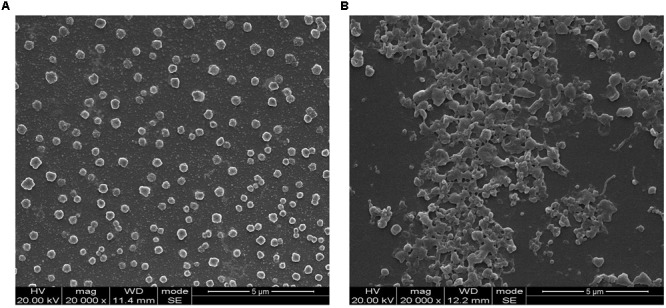
Scanning electron microscopy analysis on the *Mycoplasma bovis* 08M planktonic cells and biofilms formed on glass coverslips after 72 h of cultivation. **(A)** Planktonic cells and **(B)** biofilms.

Scanning electron microscopy analysis showed that *M. bovis* 08M formed prolific biofilms after cultivation on the coverslips for 72 h. A large number of cell clusters formed a dense net enclosed by numerous mycoplasma cells and extracellular matrix for the biofilms. On the contrary, the planktonic cells were dispersed. The morphological features of *M. bovis* biofilms exhibited significant difference from those of their planktonic counterparts (**Figure 1**).

### 2-DE Analysis of Whole-Cell Proteins From *M. bovis* Biofilms and Planktonic Cells

**FIGURE 2:**
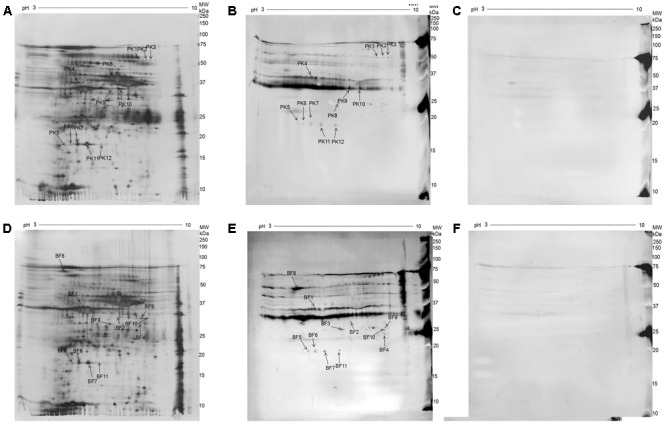
2D electrophoresis (2-DE) gel and Western blot analysis of whole-cell proteins of *M. bovis* 08M grown as planktonic cells and biofilms. **(A)** Silver-stained 2-DE gel of whole-cell proteins from *M. bovis* grown as planktonic cells (pH 3–10, 13 cm). **(B)** Western blot analysis of whole-cell proteins from *M. bovis* grown as planktonic cells using convalescent sera against *M. bovis* 08M. **(C)** Western blot analysis of whole-cell proteins from *M. bovis* grown as planktonic cells using pre-infected sera. **(D)** Silver-stained 2-DE gel analysis of whole-cell proteins from *M. bovis* grown as biofilms (pH 3–10, 13 cm). **(E)** Western blot analysis of whole-cell proteins from *M. bovis* grown as biofilm using convalescent sera against *M. bovis* 08M. **(F)** Western blot analysis of whole-cell proteins from *M. bovis* grown as biofilm using pre-infected sera.

*Mycoplasma bovis* planktonic cells (2.67 ± 0.38 × 10^8^ CFU/mL) and biofilms were harvested at 72 h, and then the whole-cell proteins were extracted. 2-DE analysis was performed to separate the whole-cell proteins of *M. bovis* planktonic cells and biofilms. This analysis was reproducible based on triplicate 2-DE gels of two group protein samples (*M. bovis* planktonic cells and biofilms) (**Supplementary Figure S1**). Approximately 1000–1300 spots were detected in 2D-E gels of *M. bovis* biofilms and planktonic cells. Approximately 596 matching protein spots were detected on the representative gels for *M. bovis* biofilms and planktonic cells with pH 3–10 IPG strips (**Figures 2A,D**), corresponding to 67.42% of the total number of coding genes of *M. bovis* 08M genome. The majority of these proteins exhibited molecular weights that ranged from 10 to 75 kDa.

**Supplementary Figure S2** shows the representative silver-stained 2D-E gel images of proteins in *M. bovis* planktonic cells and biofilms. A total of 49 significantly differentially expressed protein spots with at least a 1.5-fold abundance change were detected between *M. bovis* planktonic cells and biofilms. Compared with *M. bovis* planktonic cells, 21 (42.86%) protein spots of the differential protein spots were increased in *M. bovis* biofilms, whereas 28 (57.14%) were decreased. Among which, five spots were selected and excised from representative 2-DE gels for identification by using MALDI-TOF/TOF MS analysis, corresponding to seven proteins (**Supplementary Table S1**). Four proteins, namely, thiol peroxidase (Tpx), segregation and condensation protein B, pyruvate dehydrogenase E1 component subunit beta (PdhB), and one putative lipoprotein, were increased, and three proteins, namely, enolase, endoglucanase, and elongation factor Ts (EF-Ts), were decreased.

### Immunoblotting Analysis and Proteins Identified Using MALDI-TOF/TOF MS

A total of 12 and 11 immunoreactive protein spots that matched the protein spots detected in the 2-DE gels were observed on the immunoblot analysis of *M. bovis* planktonic cells and biofilms, respectively (**Figures 2B,E**). No specific immunoreactive protein spots reacted with the pre-infected sera for planktonic cells and biofilms (**Figures 2C,F**). MALDI-TOF/TOF MS analysis was performed to identify the protein spots. There were 12 immunoreactive protein spots for the planktonic cells and finally 8 different proteins were identified in the 11 protein spots of them. For the biofilms, 10 different proteins were identified in the 10 protein spots. For planktonic cells, six proteins corresponded to single spots and two proteins were represented by three and four isoforms, indicating the post-translational modification of these proteins. For *M. bovis* biofilms, eight proteins corresponded to single spots and two proteins were recognized by three and four isoforms. The detailed information of the identified immunoproteins for *M. bovis* planktonic cells and biofilms in this study are shown in **Tables 1, 2**. To the best of our knowledge, among these proteins, 12 proteins, including carbohydrate uptake ABC transporter-2 (CUT2) family ATP-binding protein (CUT2), endoglucanase, thiol peroxidase (Tpx), UvrABC system protein A, Pta_1, chromosome partition protein Smc, EcfA, DnaK, Era, lipoprotein, hydrolases of the HAD superfamily protein, and one uncharacterized protein, have not been reported as immunogen in *M. bovis*. Six proteins, namely, EcfA, DnaK, Era, lipoprotein, hydrolases of the HAD superfamily protein, and one uncharacterized protein, were specific to *M. bovis* biofilms. Moreover, endoglucanase, Tpx, and MilA were identified as common immunoreactive proteins in planktonic cells and biofilms.

**Table 1:** Immunoreactive proteins of *M. bovis* planktonic cells identified by MALDI-TOF/TOF MS.

Spot no.	Protein	Homology gene/	Uniprot	Theoretical	Theoretical	No. of	Protein	Protein	PSORTb	PSORTb	COG^b^
		locus tag	accession no.	protein MW	protein PI	peptides	scores	score	localization	probability	
						matched		C.I. %^a^			
PK1–PK2–PK3	Carbohydrate uptake ABC transporter-2 (CUT2) family, ATP-binding protein	MBOVPG45_0018	tr|E4PYW6|	59.27	7.79	21	324	100	Cytoplasmic Membrane	9.99	R
PK4	Endoglucanase	MBOVPG45_0256 *tpx*/	tr|A0A059Y901|	39.39	5.42	18	621	100	Cytoplasmic	7.50	EG
PK6–PK7–PK11–PK12	Thiol peroxidase (Tpx)	MBOVPG45_0640 *tuf*/	tr|A0A059Y3P0|	18.47	5.44	15	774	100	Cytoplasmic	7.50	O
PK8	Elongation factor Tu (Tuf)	MBOVPG45_0411 *uvrA*/	tr|A0A059Y8L3|	43.64	5.89	26	860	100	Cytoplasmic	9.97	J
PK9	UvrABC system protein A (UvrA)	MBOVPG45_0470 *pta-1*/	tr|E4Q030|	106.14	6.94	20	53	99.23	Cytoplasmic	7.50	L
PK10	Phosphotransacetylase (Pta_1)	MBOVPG45_0153 *milA*	tr|A0A059XYN4|	34.37	6.10	23	1,090	100	Cytoplasmic	7.50	C
PK10	Putative membrane protein (MilA)	MBOVPG45_0710 *smc*/	tr|E4Q0M5|	302.89	8.71	36	48	97.55	Unknown	–	E
PK10	Chromosome partition protein Smc (Smc)	MBOVPG45_0520	tr|A0A059XZ49|	111.20	5.74	13	47	96.92	Cytoplasmic	7.50	D

*^*a*^*C.I. %*, confidence interval.*

*^*b*^ COGs database functional categories: (C) Energy production and conversion, (D) cell cycle control, cell division, chromosome partitioning, (E) amino acid transport and metabolism, (G) carbohydrate transport and metabolism, (J) translation, ribosomal structure and biogenesis, (L) replication, recombination and repair, (O) post-translational modification, protein turnover, chaperones, (P) inorganic ion transport and metabolism, (R) general function prediction only.*

**Table 2:** Immunoreactive proteins of *M. bovis* biofilms identified by MALDI-TOF/TOF MS.

Spot no.	Protein	Homology gene/locus tag	Uniprot accession no.	Theoretical protein MW	Theoretical protein PI	No. of peptides matched	Protein scores	Protein score CI %^a^	PSORTb localization	PSORTb probability	COG^b^
BF1	Endoglucanase	MBOVPG45_0256	tr|A0A059Y901|	39.39	5.42	18	621	100	Cytoplasmic	7.50	EG
BF3	Energy-coupling factor transporter ATP-binding protein EcfA	*ecfA*/ MBOVPG45_0295	tr|A0A059XZR6|	29.41	6.02	7	95	100	Cytoplasmic	7.50	PR
BF3	Putative membrane protein	MBOVPG45_0710	tr|E4Q0M5|	302.89	8.71	43	64	99.94	Unknown	–	E
BF4–BF9–BF10	Pyruvate dehydrogenase E1 component subunit alpha (PdhA)	*pdhA*/ MBOVPG45_0104	tr|A0A059XYH6|	41.33	6.13	10	298	100	Cytoplasmic	9.97	C
BF5–BF6–BF7–BF11	Thiol peroxidase (Tpx)	*tpx*/ MBOVPG45_0640	tr|A0A059Y3P0|	18.47	5.44	13	906	100	Cytoplasmic	7.50	O
BF8	Chaperone protein DnaK (DnaK)	*dnaK*/ MBOVPG45_0160	tr|A0A059Y7X3|	65.30	4.95	28	692	100	Cytoplasmic	9.97	O
BF8	GTPase Era (Era)	*era*/ MBOVPG45_0126	tr|A0A059Y7U9|	32.94	8.54	13	54	99.31	Cytoplasmic membrane	8.78	J
BF8	Lipoprotein	MBOVPG45_0215	tr|A0A059XZZ3|	48.62	9.16	13	47	96.92	Unknown	–	R
BF9	Hydrolases of the HAD superfamily protein	MBOVPG45_0665	tr|A0A059Y395|	34.76	6.62	18	660	100	Cytoplasmic	7.50	HR
BF10	Uncharacterized protein	MBOVPG45_0718	tr|A0A059Y955|	34.91	8.13	14	61	99.87	Cytoplasmic	7.50	X

*^*a*^ C.I. %, confidence interval.*

*^*b*^ COGs database functional categories: (C) Energy production and conversion, (D) cell cycle control, cell division, chromosome partitioning, (E) amino acid transport and metabolism, (G) carbohydrate transport and metabolism, (H) co-enzyme transport and metabolism, (J) translation, ribosomal structure and biogenesis, (L) replication, recombination and repair, (O) post-translational modification, protein turnover, chaperones, (P) inorganic ion transport and metabolism, (R) general function prediction only, (X) mobilome: prophages, transposons.*

### Bioinformatics Analysis

**FIGURE 3:**
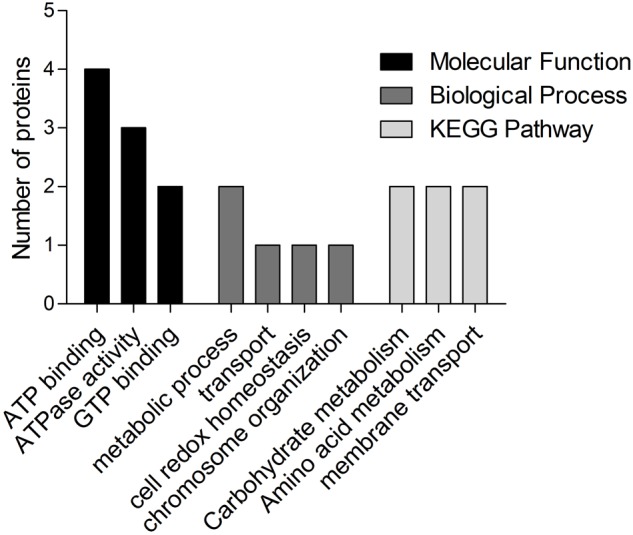
Most enriched categories of GO in terms of biological process, molecular function, and KEGG pathways of the identified immunoproteins.

**FIGURE 4:**
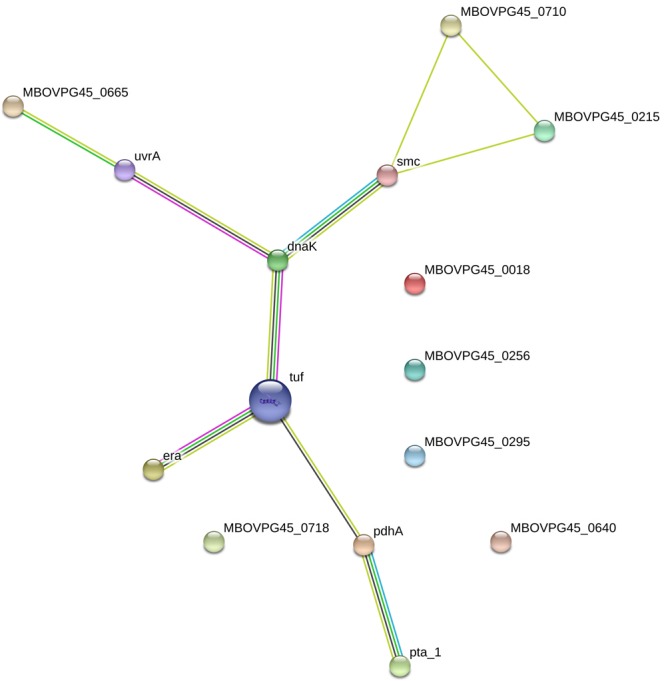
STRING network generated with the available immunoproteins from **Tables 1, 2**.

Gene Ontology and KEGG pathway analyses of the identified proteins are shown in **Figure 3**. For the biological process of GO, various biological processes, which mainly included metabolic process (GO:0008152), transport (GO:0006810), cell redox homeostasis (GO:0045454), and chromosome organization (GO:0051276), were observed. The most representative molecular functions of these proteins mainly included ATP binding (GO:0005524), oxidoreductase activity (GO:0016491), and GTP binding (GO:0005525). These proteins were largely involved in metabolic pathways, such as metabolism, carbohydrate metabolism, and amino acid metabolism. For the COG analysis of the identified immunoproteins, various categories, including amino acid transport and metabolism, general function predicted only, post-translational modification, protein turnover, and chaperone, energy production and conversion, translation, ribosomal structure and biogenesis, were also involved (**Tables 1, 2**). PSORTb v.3.0 database was used to predict the subcellular localization of the identified proteins. Results showed that CUT2 and Era were associated to the membrane. The remaining 11 immunoproteins were predicted to display cytoplasmic localizations (**Tables 1, 2**). STRING tool was used to predict the potential protein–protein interaction of the identified immunoproteins in **Tables 1, 2** using *M. bovis*-type strain PG45 database as the default reference. The STRING network generated with the immunoproteins is shown in **Figure 4**. Results showed that the most significant proteins that are likely to interact are DnaK and elongation factor Tu (Tuf), suggesting that these proteins may function together.

### Confirmation of the Immunogenicity for Selected Proteins

**FIGURE 5:**
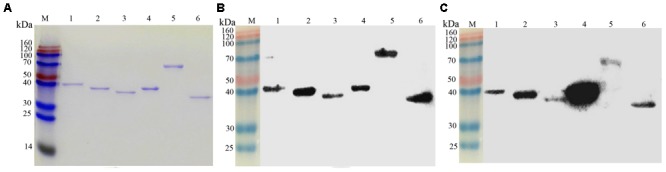
Analysis of six recombinant expressed proteins. **(A)** SDS-PAGE of six purified recombinant expressed proteins. **(B)** Western blot analysis of recombinant expressed proteins using convalescent sera against *M. bovis* 08M. **(C)** Western blot analysis of recombinant expressed proteins using bovine immune-sera against *M. bovis*. “M” represents the protein markers, and the numbers on the left indicate the molecular weight standards (kDa). Lines 1–6: Endoglucanase, Era, hydrolases of the HAD superfamily protein, Pta_1, DnaK, and EcfA.

To confirm the immunogenicity of endoglucanase, Era, hydrolases of the HAD superfamily protein, Pta_1, DnaK, and EcfA, the recombinant selected proteins were successfully cloned and expressed in *E. coli* BL21(DE3) cells (**Figure 5A**). Western blot analysis showed that the purified recombinant proteins can react with bovine-immune and convalescent sera against *M. bovis* (**Figures 5B,C**), but not with healthy bovine sera (data not shown). The results indicated the good immunogenicity of these identified proteins. A very big spot was observed for Pta_1 (**Figure 5C**) upon immunoblotting using the sera obtained from immunized animals, demonstrating that among the selected proteins, Pta_1 had the highest reactivity with bovine-immune sera against *M. bovis*.

## Discussion

*Mycoplasma bovis* infection has become a major threat to the cattle industry in many countries. In the past few decades, considerable efforts focused on identifying the immunogenic proteins of *M. bovis* to develop diagnostic kits and vaccines to control its infection (Perez-Casal et al., 2017). Several immunogenic proteins were identified in different components of *M. bovis* planktonic cells (Sun et al., 2014; Khan et al., 2016). To the best of our knowledge, the immunogenic component of *M. bovis* biofilm remains unknown. In the present study, the immunogenic proteins of *M. bovis* biofilms were identified and compared with their planktonic counterparts.

*Mycoplasma bovis* can form biofilms *in vitro* (Mcauliffe et al., 2006); however, whether the biofilm formation can occur *in vivo* remains unknown. In the present study, whole-cell proteins of *M. bovis* biofilms and planktonic cells were prepared *in vitro*. 2-DE, immunoblotting, and MALDI-TOF/TOF MS were performed to identify the immunogens of *M. bovis* in these two growth modes. The early generation of a virulent Chinese strain 08M was used to avoid antigenic variation. Moreover, whole-cell proteins were prepared as protein samples for 2-DE instead of membrane proteins to prevent the loss of important cytoplasmic antigen proteins. Report showed that some additional promising antigens for diagnostics could be obtained in the membrane fraction of *M. bovis* planktonic cells (Khan et al., 2016). Thus, identifying the membrane immunoproteomic surface of *M. bovis* biofilms and planktonic cells will be useful and should be studied further.

*Mycoplasma bovis* biofilms and planktonic cells presented different antigen profiles. This phenomenon was also observed in *Streptococcus pneumoniae*, and it may be related to protein expression (Sanchez et al., 2011). Significantly differentially expressed proteins were found between *M. bovis* planktonic cells and biofilms in the 2D-E analysis. For example, Tpx, which is related to oxidative stress response, was increased in *M. bovis* biofilms. Kalmokoff et al. (2006) used 2D-E to reveal that Tpx was up-regulated in *Campylobacter jejuni* biofilms. Enolase and EF-Ts, which are involved in glycolysis and carbohydrate metabolism, and protein synthesis, respectively, were down-regulated in *M. bovis* biofilms; this finding was also described in *Lactobacillus plantarum* (De Angelis et al., 2015) biofilms compared to planktonic cells. In the present study, endoglucanase, Tpx, and MilA were identified in planktonic and biofilm-grown *M. bovis*. Common immunoreactive proteins may be promising candidates for the development of a vaccine that can prevent biofilm formation and *M. bovis* infection. A total of 12 novel immunogens may also be molecular candidates for the development of improved diagnostics and vaccines to control *M. bovis* infections. The remaining pyruvate dehydrogenase E1 component subunit alpha (PdhA) (Sun et al., 2014), Tuf (Khan et al., 2016), and MilA (Wawegama et al., 2014) were previously reported as antigens in *M. bovis*. Pta-1, which is involved in the synthesis of acetyl-CoA from acetate, was an immunogen in Mmc (Corona et al., 2013) but is not reported as an immunogen in *M. bovis.* DnaK was identified as an immunogenic protein in several mycoplasma species in planktonic mode, such as MmmSC (Jores et al., 2009), Mccp (Zhao et al., 2012), and Mmc (Churchward et al., 2015). However, DnaK is not reported as an immunogen in *M. bovis* planktonic cells, to the best of our knowledge. This discrepancy might due to the different species of *Mycoplasma* or the serum used. Notably, DnaK was identified as immunogenic in the *M. bovis* biofilms, same with other bacteria biofilms, such as *S. pneumoniae* (Sanchez et al., 2011), *Streptococcus suis* (Wang et al., 2012), and *Staphylococcus epidermidis* (Carvalhais et al., 2015). Tuf promotes the GTP-dependent binding of aminoacyl-tRNA to the A-site of ribosomes during protein biosynthesis. It was shown to be an highly immunogenic protein in many *Mycoplasma* species (Jores et al., 2009; Churchward et al., 2015), and was also identified in *M. bovis* planktonic cells in this study. Moreover, Tuf was predicted by PSORTb database to be cytoplasmically localized in many *Mycoplasma* species (Churchward et al., 2015; Khan et al., 2016). In *M. pneumoniae*, Tuf was observed to be surface-localized protein that can mediate binding to fibronectin (Dallo et al., 2002). Furthermore, DnaK was reported to be a surface-localized protein that can mediate binding to host receptors (Boulanger et al., 1995). In this study, DnaK and Tuf were predicted to be cytoplasmically localized in *M. bovis* and appeared to be the most interactive immunoreactive and may be related to *M. bovis* biofilm formation. DnaK and Tuf were reported to be differently expressed in biofilms and planktonic cells for some bacteria, such as *Streptococcus equi* ssp. *zooepidemicus* (Yi et al., 2016) and *L. plantarum* (De Angelis et al., 2015), and may be related to biofilm formation. The subcellular localization and role of these immunoreactive proteins in the *M. bovis* biofilm formation should be investigated further.

Several antigens, such as pMB67, glyceraldehyde-3-phosphate dehydrogenase, the pyruvate dehydrogenase beta subunit, and other antigens identified in previous studies (Sachse et al., 1993; Robino et al., 2005; Sun et al., 2014; Khan et al., 2016), were not detected in the planktonic cells in the present study. This phenomenon was previously described (Khan et al., 2016) and might be due to the differences in methods and antiserum selection. Moreover, antigen profiles may exhibit several differences due to various strains, such as antigen variation, as reported previously in *R. anatipestifer* (Hu et al., 2012). The selection of antiserum is crucial in the screening of immunogenic proteins. Antisera from natural and experimental infections may present different antigen profiles for *M. bovis* because of different antibody titers, doses, and courses in natural and experimental infections. In the present study, immunoblotting with different sera obtained from cattle experimentally infected with *M. bovis* was performed in the preliminary study. Finally, pooled sera of 46 dpi were selected to obtain considerable amount of immunogenic proteins.

Endoglucanase, Era, hydrolases of the HAD superfamily protein, Pta_1, DnaK, and EcfA that were identified in the present study were selected to express the recombinant protein in *E. coli*, to be further evaluated as molecular candidates for diagnostics or vaccines. The results showed that these selected proteins can all react with bovine-immune and convalescent sera against *M. bovis.* Pta_1 had the highest reactivity with sera obtained from immunized animals among the selected proteins, possibly because Pta_1 was a dominant immunoreactive protein in *M. bovis* and was also described in Mmc (Churchward et al., 2015). The selected proteins may be as novel molecular candidates of diagnostics or vaccines but this finding needs further evaluation.

## Conclusion

In the present study, immunoproteomic analysis was used to compare the antigen profiles of *M. bovis* biofilms and planktonic cells reacted with bovine convalescent sera. Differential immunoreactivity to bovine convalescent serum between *M. bovis* biofilms and planktonic cells were observed. A total of 12 novel immunoreactive proteins were identified. Most of the identified proteins were cytoplasmic proteins that were mainly involved in transport and metabolism. DnaK and Tuf appeared to be the most interactive immunoreactive proteins and may be related to *M. bovis* biofilm formation. Six proteins had potential as serodiagnostic antigens but still needs further evaluation. These results lay the foundation for the understanding of the host response to *M. bovis* in two growth modes and may facilitate the development of novel molecular candidates for the improved diagnostics and vaccines for controlling *M. bovis* infections.

## Author Contributions

SC conceived and designed the experiments. SC, HH, PZ, WJ, and ML performed the experiments: SC and HH wrote the manuscript and analyzed the data. YC and YL contributed reagents/materials/analysis tools. SC, HH, and YC critically revised the manuscript. YC and YL supervised all work. All authors read and approved the final version of the manuscript.

## Conflict of Interest Statement

The authors declare that the research was conducted in the absence of any commercial or financial relationships that could be construed as a potential conflict of interest. The reviewer SA and handling Editor declared their shared affiliation.

## Footnotes

1 http://geneontology.org/

2 http://www.ncbi.nlm.nih.gov/COG/

3 http://www.genome.jp/kegg/

4 http://www.psort.org/

5 http://www.string-db.org/

## Supplementary Material

FIGURE S1 2-DE gel images of proteins in *M. bovis* planktonic cells and biofilms in triplicates.

FIGURE S2 Image of proteins of representative 2D-E gel in *M. bovis* planktonic cells and biofilms. **(A)** The representative silver-stained 2D-E gel image of *M. bovis* planktonic cells and biofilms. Differentially expressed protein spots were circled with numbers by using ImageMaster^TM^ 2D Platinum 5.0 software. **(B)** Expanded region of differentially expressed proteins spot with circles.

TABLE S1 Selected differentially expressed proteins spots identified by MALDI-TOF/TOF MS between *M. bovis* planktonic cells and biofilms.

## Abbreviations

2D: two dimensional
2-DE: two dimensional gel electrophoresis
CHAPS: 3-[(3-cholamidopropyl)dimethylammonio]-1-propanesulfonate
DTT: dithiothreitol
GO: Gene Ontology
IEF: isoelectric focusing
IPG: immobilized pH gradient
KEGG: Kyoto Encyclopedia of Genes and Genomes
MALDI-TOF/TOF MS: matrix-assisted laser desorption/ionization time-of-flight tandem mass spectrometry
MS: mass spectrometry
PBS: phosphate buffered saline
PPLO broth: pleuropneumonia-like organism broth
PVDF: polyvinylidene fluoride
SDS: sodium dodecyl sulfate
SDS-PAGE: sodium dodecyl sulfate polyacrylamide gel electrophoresis
SEM: scanning electron microscopy
TBST: tris buffered saline with tween
