# Supplementary figures and images for: Differential Immunoreactivity to Bovine Convalescent Serum Between Mycoplasma bovis Biofilms and Planktonic Cells Revealed by Comparative Immunoproteomic Analysis
Source: Front Microbiol. 2018 Mar 5;9:379. doi: 10.3389/fmicb.2018.00379 (PMC5844979; doi:10.3389/fmicb.2018.00379)

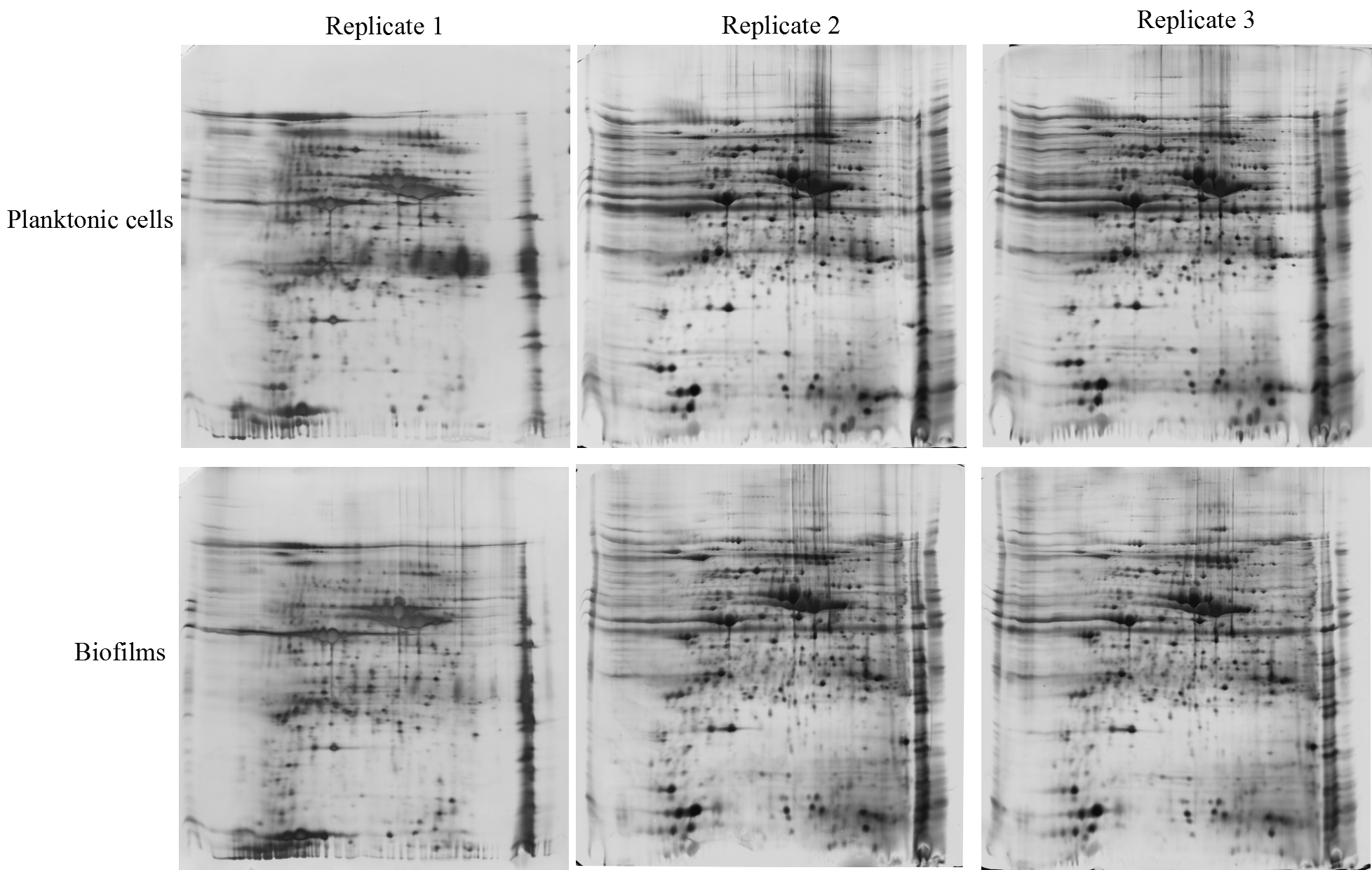

Supplement: FIGURE S1 — 2-DE gel images of proteins in M. bovis planktonic cells and biofilms in triplicates. [file Image_1.TIF]

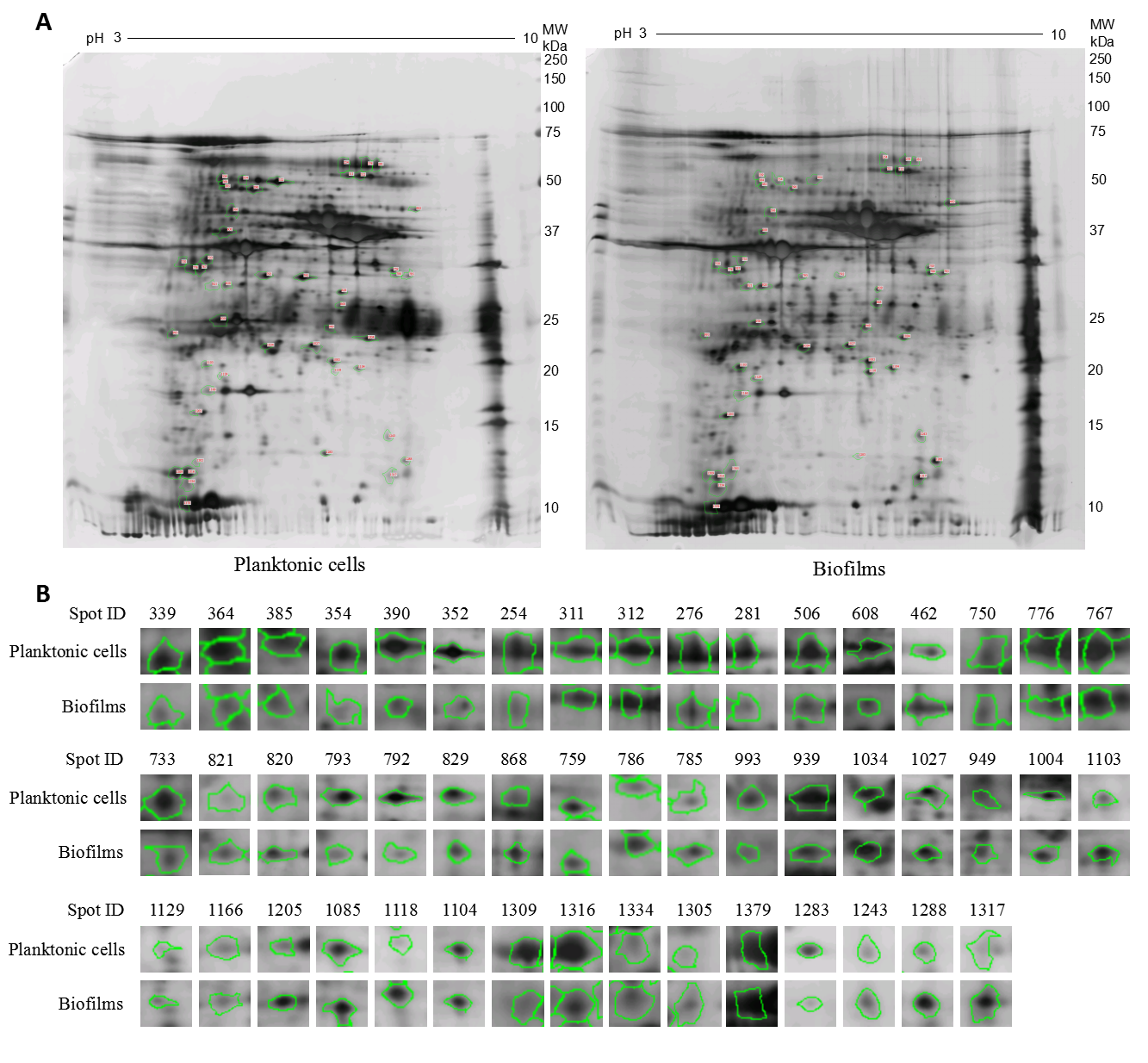

Supplement: FIGURE S2 — Image of proteins of representative 2D-E gel in M. bovis planktonic cells and biofilms. (A) The representative silver-stained 2D-E gel image of M. bovis planktonic cells and biofilms. Differentially expressed protein spots were circled with numbers by using ImageMasterTM 2D Platinum 5.0 software. (B) Expanded region of differentially expressed proteins spot with circles. [file Image_2.TIF]
