# Supplementary material for: Differential Immunoreactivity to Bovine Convalescent Serum Between Mycoplasma bovis Biofilms and Planktonic Cells Revealed by Comparative Immunoproteomic Analysis
Source: Front Microbiol. 2018 Mar 5;9:379. doi: 10.3389/fmicb.2018.00379 (PMC5844979; doi:10.3389/fmicb.2018.00379)
Supplement: TABLE S1 — Selected differentially expressed proteins spots identified by MALDI-TOF/TOF MS between M. bovis planktonic cells and biofilms. [file Table_1.DOC]

Table S1 Selected differentially expressed proteins spots identified by MALDI-TOF/TOF MS between *M. bovis* planktonic cells and biofilms

| Spot ID^a^. | Protein | Homology Gene/ locus tag | Uniprot  Accession no. | Theoretical Protein MW | Theoretical Protein PI | No. of peptides matched | Protein  Scores | Protein Score  C. I. %^b^ | T test  P value | Fold change^c^  Increase (+)/ decrease (-) | COG^d^ |
| --- | --- | --- | --- | --- | --- | --- | --- | --- | --- | --- | --- |
| 390 | Enolase | *eno/*  MBOVPG45_0409 | tr|A0A059Y8Q7| | 49.57 | 5.45 | 20 | 691 | 100 | 0.003 | -3.81 | G |
| 608 | Endoglucanase | MBOVPG45_0256 | tr|A0A059Y901| | 39.39 | 5.42 | 18 | 621 | 100 | 0.041 | -2.74 | EG |
| 767 | Elongation factor Ts (EF-Ts) | *tsf/*  MBOVPG45_0558 | tr|E4Q0A9| | 32.67 | 5.23 | 17 | 503 | 100 | 0.027 | -1.58 | J |
| 1034 | Segregation and condensation protein B (ScpB) | *scpB*  K668_03825 | tr|A0A059Y4R5| | 21.79 | 5.65 | 17 | 573 | 100 | 0.038 | +1.52 | K |
| 1034 | Pyruvate dehydrogenase E1 component subunit beta (PdhB) | *pdhB/*  MBOVPG45_0105 | tr\|E4PZ47\| | 36.18 | 5.44 | 7 | 259 | 100 |  |  | C |
| 1034 | Putative lipoprotein | MBOVPG45_0112 | tr\|E4PZ53\| | 110.32 | 8.63 | 21 | 50 | 98.34 |  |  | - |
| 1166 | Thiol peroxidase (Tpx) | *tpx*/  MBOVPG45_0640 | tr|A0A059Y3P0| | 18.47 | 5.44 | 15 | 774 | 100 | 0.009 | +1.52 | O |

^a^Spot ID, refer to the spot number given in Figure S2.

^b^C.I.%: Confidence interval

^c^Fold change calculated the overlapping measures ratio of corresponding spots by using Image Master 2D™ Platinum software. Increase: The abundance level of *M. bovis* biofilms is higher than planktonic cells. Decrease: The abundance level of *M. bovis* biofilms is lower than planktonic cells.

^d^COGs database functional categories: (C) Energy production and conversion, (E) amino acid transport and metabolism, (G) carbohydrate transport and metabolism, (J) translation, ribosomal structure and biogenesis, (K) transcription, (O) post-translational modification, protein turnover, chaperones.
